# A urinary DNA methylation assay using two genes enables noninvasive detection and prognostic prediction in urothelial carcinoma

**DOI:** 10.1038/s41598-025-14646-0

**Published:** 2025-08-25

**Authors:** Dong Wang, Lu Ai, Hongxia Tan, Yan Yan, Hui Ye, Meifang He, Chun-Hui Huang, Peisong Chen, Yanping Liang, Ruizhi Wang

**Affiliations:** 1 Department of Laboratory Medicine, The First Affiliated Hospital of Sun Yat-sen University, No. 58, Zhongshan 2nd Road, Guangzhou, 510080 China; 2 Department of Laboratory Medicine, Guangxi Hospital Division of the First Affiliated Hospital, Sun Yat-Sen University, Naning, 530028 China; 3 Department of Laboratory Medicine, The Third Affiliated Hospital of GanNan Medical University, No. 46, Jingjiu Road, Ganzhou, 341000 China; 4 Laboratory of General Surgery, The First Affiliated Hospital of Sun Yat-sen University, No. 58, Zhongshan 2nd Road, Guangzhou, 510080 China; 5 Department of Clinical Laboratory, Guangxi Academy of Medical Sciences, The People’s Hospital of Guangxi Zhuang Autonomous Region, No.6 Taoyuan Road, Nanning, 530000 China; 6 Advanced Medicine Technology Center, The First Affiliated Hospital, Zhongshan School of Medicine, Sun Yat-sen University, Guangzhou, 510080 China

**Keywords:** Urothelial carcinoma, DNA methylation, Urine, Noninvasive detection, Cancer screening, Tumour biomarkers, Urological cancer

## Abstract

The incidence of urothelial carcinoma (UC) ranks second among all urological cancers, accounting for over 90% of malignant tumors in bladder. Patients diagnosed with UC experience a lower quality of life due to rapid progression of the disease. Early and non-invasive detection is curial for diagnosing UC and improving patient outcomes. This study aimed to develop and validate a DNA methylation assay for the early detection and monitoring for UC, with a focus on its diagnostic and prognostic implications. The DNA methylation assay with real-time methylation specific polymerase chain reaction (RT-MSP) technique measures the methylation level of SOX1-OT and HIST1H4F in urine samples. A cohort comprising 436 patients diagnosed with UC or other urologic disease as well as 79 healthy patients was retrospectively utilized to evaluate this assay. Furthermore, UC patients who underwent surgery are included to assess its ability to detect recurrence. The DNA methylation assay demonstrated significantly increased methylation level in tumor tissues and a high positive rate in urine samples from UC patients. The methylation profile effectively distinguishes UC from other non-UC urologic disease, exhibiting a high sensitivity (85.2%) and specificity (90.0%) for UC diagnosis. Furthermore, the assay showed promising ability in differentiating UC patients based on tumor grade, malignancy potential, and disease stage. Additionally, the DNA methylation assay demonstrated a superior ability to detect UC recurrence with a high area under curve (AUC) of 0.979. This research proposes a novel DNA methylation assay with urine as sample for detection, representing a cost-efficient and non-invasive method for diagnosing and monitoring UC disease.

**Supplementary Information:**

The online version contains supplementary material available at 10.1038/s41598-025-14646-0.

## Introduction

Urothelial carcinoma (UC) is the second most prevalent malignant tumor in urology, posing a significant threat to the quality of life and survival of patients, especially in male patients^1,2^. Bladder urothelial carcinoma accounts for over 90% of UC cases, which accounts for 6% of all new cancer cases in male, and can be categorized into non-muscle-invasive (NMIBC) and muscle-invasive (MIBC) subtypes. About 70% of UC subtypes are non-muscle-invasive, and they can be further divided into low-, intermediate- and high-risk groups based on a scoring system regarding six clinical and pathological features (number of tumors, tumor size, prior recurrence rate, T category, carcinoma in situ, and grade)^3,4^. Generally, the prognosis of MIBC, no matter it is de novo or progressed from NMIBC, is worse than NMIBC. Despite prompt and standard treatments, non-muscle-invasive UC patients face a high recurrence rate of 70%. Eventually, approximately 15% of these recurrent patients will progress to higher stages and grades^5^. Because non-muscle-invasive disease demonstrates a superior prognosis compared to muscle-invasive disease^6^, routine screening is important to detect and treat non-muscle-invasive disease in a timely manner, in order to achieve best outcome for patient.

UC often presents with typical manifestations of urinary tract diseases and lacks specific clinical features. Hematuria, the detection of red blood cells in urine, is one of the most common presentations in urologic clinical practice^7^, and up to 20% of patients with gross hematuria are diagnosed with an underlying urological malignant condition^8^. Currently, cystoscopy combined with lesion biopsy is considered the most accurate method for diagnosing UC^9^. However, this manipulation is invasive, costly, and may occasionally miss the lesion site. In recent years, urine-based detection methods have provided a convenient and cost-effective diagnostic option for urological malignancies. For example, frequent mutations of the PLEKHS1 promoter in urine tumor DNA have been observed in bladder cancer patients, serving as a diagnostic marker^10^. Additionally, a protocol utilizing an automated device for urine cancer cell detection in bladder cancer patients has shown promising result^11^. In summary, more efficient methods are being developed to achieve superior balance among cost, time and accuracy.

Recently, DNA methylation assays have emerged as a promising target in the field of urine screening for UC. Early DNA methylation changes are considered as crucial regulators in tumorigenesis^12,13^, and their stability allows for detection in urine. Up to now, several DNA methylation assays have been studied in UC, with variable consistency and accuracy^14–16^. Therefore, there is a need for further efforts to develop a reliable and cost-effective DNA methylation assay for UC patients.

This study organized a cohort of patients, collected urine samples and clinical information, and tested a DNA methylation assay based on two targets, SOX1-OT and HIST1H4F. These two biomarkers were chosen based on thorough literature review and preliminary samples screening from our hospital. HIST1H4F is a histone-related gene and responsible for histone H4 expression, while the biological function of SOX1-OT is still unknown^15,17^. The results demonstrated excellent diagnostic and differential power, providing novel insights for non-invasive UC detection and highlighting the great potential of urine biomarkers in UC diagnosis.

## Materials and methods

### Participants

This study was approved by the Human Ethics Committee of the First Affiliated Hospital of Sun Yat-Sen University, which follows the rules of Helsinki Declaration. The ethical code of our study is [2022]549. Written informed consents were obtained from all participants. All the participants were enrolled consecutively and randomly from the First Affiliated Hospital of Sun Yat-Sen University between January 2021 and December 2023. Among them, 436 patients suffering from urological symptoms were newly diagnosed with urothelial carcinoma (135 patients), renal cell carcinoma (39 patients), prostate cancer (45 patients), or other urologic tumors (161 patients, including adrenal adenoma, adrenal myelolipoma, pheochromocytoma non-Hodgkin lymphoma, renal papillary carcinoma, renal chromophobe, renal angiomyolipoma, bladder paraganglioma, bladder neuroendocrine tumors and metastatic tumors) as well as benign non-tumor urologic diseases (56 patients, including chronic pyelonephritis, chronic cystitis, hydronephrosis, duplex kidneys, bladder endometriosis, bladder diverticula and normal bladder). All diagnoses were confirmed through pathology determination via either surgical pathology or cystoscopy biopsy. Moreover, additional 179 follow-up patients diagnosed with UC who underwent transurethral resection were recruited, with a median follow-up time of 12.3 months (1-120.4 months). A group of 79 healthy volunteers were also recruited as healthy controls. For UC, tumor staging and grading were determined according to the World Health Organization (WHO) 2004/2016 (LG-HG) classification systems. Specifically, for the 135 UC patients in the cohort, 63 patients had non-invasive disease and 72 patients had invasive disease. These patients could be further stratified into four groups: papillary urothelial neoplasm of low/non-malignant potential (10 patients), low-grade non-invasive papillary urothelial carcinoma (16 patients), low-grade invasive papillary urothelial carcinoma (4 patients), high grade non-invasive papillary urothelial carcinoma (24 patients) and high grade invasive papillary urothelial carcinoma (81 patients).

### Samples

For the newly diagnosed or UC-recurrent patients, urine samples (10 mL per patient) were collected prior to cystoscopy or surgery. For healthy and UC-follow-up participants, morning urine (10 mL per patient) was collected. Cell debris and pellets along with 300 µL of urine from the urine specimens were obtained by centrifugation at 5500 rpm for 10 min and immediately stored at −80 °C before DNA extraction.

Tumor specimens of bladder urothelial carcinoma were obtained from the Department of Urology, the First Affiliated Hospital of Sun Yat-sen University. Fresh tumor and para-cancerous normal tissues were dissected and stored at ‐80 °C before DNA extraction for DNA methylation-specific PCR analysis. The para-cancerous normal tissues were collected at least 5 cm away from the tumor site, in order to minimize the contamination form tumor.

### DNA extraction and real-time methylation specific polymerase chain reaction (RT-MSP)

The urine sediment was thoroughly resuspended and the genomic DNA was extracted from the urinary sediment using the MagPure Nucleic Acid Kit (Chaozhou Hybribio Biochemistry, China) following the manufacturer’s strict guidelines. Subsequently, the NanoDrop 500 spectrophotometer (Hangzhou Allsheng Instruments, China) was utilized to ensure a 260/280 ratio within the range of 1.4–2.1 and a minimum DNA concentration of 10 ng/µL for all DNA samples.

The methylation level of SOX1-OT and HIST1H4F were assessed using DNA real-time methylation-specific PCR (RT-MSP). Following DNA extraction, bisulfite conversion was meticulously performed on 500 ng DNA of each sample using the DNAm Bisulfite Conversion Kit (Guangzhou Hybribio Medicine Technology, China). The resulting product was subsequently eluted in 40µL of M-Elution Buffer and stored at a −80 °C to ensure preservation.

RT-MSP analysis was carried out using a thermal cycler (Applied Biosystems 7500, Thermo Fisher, USA) at 95 °C for 10 min, 45 cycles at 95 °C for 20 s, 60 °C for 30 s and 72 °C, in conjunction with the SOX1-OT and HIST1H4F Methylation Real-time PCR Kit provided by Guangzhou Hybribio Medicine Technology Ltd., with ACTB serving as the reference gene. Ct values for SOX1-OT, HIST1H4F1, and ACTB were determined for each sample. A Ct value for ACTB falling within the range of 15 to 35 was considered acceptable; otherwise, the sample would be subjected to retesting. A Ct value below 38 for SOX1-OT or HIST1H4F indicated a positive methylation status for this kit. ΔCt value (raw CT values of methylated SOX1-OT or HIST1H4F1 subtracted by the corresponding ACTB CT values) was used in comparison between tumor and paratumor samples, while raw CT value (unadjusted raw CT values of methylated SOX1-OT and HIST1H4F1) was used in comparison between different urine samples. Larger ΔCt or CT values indicate lower methylation level. The test result was classified as positive if either one or both of the genes exhibited positive methylation status. The methylation primer sequences used for this study are described in Supporting Table 1.

**Table 1:** Screening of candidate CpG sites for urothelial carcinoma detection.

gene ID	probe ID	UC patients (10)						benign urological diseases (5)		Healthy control (5)	
		Tumor		paratumor		Urine		Urine		Urine	
		positive %	ΔCt value	positive %	ΔCt value	positive %	Ct value	positive %	Ct value	positive %	Ct value
HIST1H4F	cg10723962	50	5.98(4.68–6.97)	20	11.14(7.99–15.84)	40	35.67(33.45–36.77)	0	/	20	35.93(34.21–37.65)
	cg21425842, cg08260959	100	5.68(3.13–8.28)	100	10.17(7.26–20.64)	80	32.79(31.10-34.82)	0	/	0	/
SOX1-OT	cg11437784	40	4.55(2.22–6.15)	10	7.24	30	34.19(32.41–36.51)	0	/	0	/
	cg15736169, cg03898631	100	4.06(1.92–8.09)	100	7.80(5.24–11.38)	80	32.62(31.72–35.16)	0	/	0	/
NRN1	cg11564981	70	3.85(2.60–5.30)	20	6.28(5.55-7.00)	50	34.64(32.55–36.14)	0	/	0	/
POU4F2	cg02610222	30	8.54(6.57–10.87)	0	/	20	37.08(36.51–37.65)	0	/	0	/
	cg24199834	60	5.76(3.11–10.79)	30	8.86(8.44–9.14)	40	33.82(31.66–35.59)	0	/	0	/
Vim	cg11973177	80	6.87(5.27–8.64)	30	9.98(5.44–13.63)	80	31.58(29.74–36.46)	0	/	30	33.89(31.27–35.27)
	cg21425842	100	4.11(2.14–5.70)	100	7.27(4.97–14.14)	70	31.66(30.02–33.96)	60	33.13(31.21–35.64)	60	33.01(30.98–35.01)
Onecut2	cg02250594	100	7.49(4.42–10.97)	40	9.74(7.87–12.54)	80	29.16(24.82–38.34)	20	33.14(32.96–33.32)	20	34.03(33.19–34.87)
	cg23757446	100	4.63(2.84–8.09)	100	8.94(6.01–13.05)	60	35.47(33.16–37.12)	40	36.97(34.45–38.87)	40	36.87(34.33–38.24)
Twist1	cg26818735, cg26312150	60	3.83(1.84–6.38)	20	7.01(5.32–6.88)	30	39.92(39.50-40.34)	0	/	0	/

### Statistical analyses

Statistical analyses and graphs construction were performed using GraphPad Prism 7. Kruskal-Wallis test was used to compared continuous variables among different groups (> 2 groups, non-parametric). Chi-square test (or Fisher’s exact test if needed) was used to test the significance between categorical variables. A *P* < 0.05 was considered significant (*P* < 0.05 for *; *P* < 0.01 for **, *P* < 0.001 for ***).

For sample size determination, the expression for the estimated sample size *n* is as follow:

$$n = z^2{}_{1 - \alpha/2} \times P \times (1 - P) / d2,$$

where *z* represents the number of standard errors away from the mean, *P* is the unknown prevalence of the disease, and d is the precision level. The values of *P* were estimated by taking reference from the proportion of urothelial carcinoma in patients with urological symptoms at the First Affiliated Hospital of Sun Yat-Sen University (20%−40%), and a value of 30% was used. While the absolute precision is desired to be set at 5.0% with 95% level of significance, the estimated sample size would be 1.962 × (0.3) × (1–0.3)/0.052 = 323. Considering a dropout rate of 20%, the expected sample size was 403 and at last a total of 436 patients were enrolled.

## Results

### CpG site selection in the methylation assay

In order to develop the methylation assay for detecting urothelial carcinoma, we conducted a screening of 14 CpG sites in 7 genes (HIST1H4F, SOX1-OT, Vim, Onecut2, Twist1, NRN1, POU4F2) based on the literature review^15,17–20^. These sites were assessed for methylation in tumor tissues, para-cancerous tissues, and preoperative urine samples from 10 patients with UC, along with urine samples from 5 patients with benign urological diseases (including urinary tract infection, cystitis and benign prostatic hyperplasia) and 5 healthy individuals. The results revealed significantly increased methylation of two CpG sites in HIST1H4F and SOX1-OT in tumor tissues, with the highest positive rate in urine samples from UC patients and the lowest positive rate in non-tumor patients (Table 1). Therefore, the methylation assay based on this two-gene DNA methylation combination was subsequently studied and validated.

### Assay performance in distinguishing UC patients from healthy individuals and patients with non-UC urologic diseases

To further verify the diagnostic performance of the assay, we collected 436 urine samples. These samples consisted of a variety of clinical conditions, including patients with newly diagnosed UC (*n* = 135), clear cell renal cell carcinoma (*n* = 39), prostate cancer (*n* = 45), other tumors of the urinary system (*n* = 161), other benign urological conditions (*n* = 56) and healthy peoples (*n* = 79) (**Supporting** Table 2). All samples were subjected to DNA methylation assay and the positive rates were compared between different groups.

**Table 2:** Analyses for the assay performance in the UC detection.

	All UC		High-grade UC		Low-grade UC		Non-invasive UC		Invasive UC	
	vs.		vs.		vs.		vs.		vs.	
	**Healthy**	**Non-UC**	**Healthy**	**Non-UC**	**Healthy**	**Non-UC**	**Healthy**	**Non-UC**	**Healthy**	**Non-UC**
**Sensitive**,** %**	85.2	85.2	92.4	92.4	80.0	80.0	70.0	70.0	94.1	94.1
**Specificity**,** %**	100.0	90.0	100.0	90.0	100.0	90.0	100.0	89.4	100.0	89.4
**PPV**^**a**^, **%**	100.0	79.3	100.0	76.4	100.0	34.8	100.0	52.2	100.0	74.1
**NPV**^**b**^, **%**	79.8	93.1	90.8	97.1	95.2	98.5	84.0	94.8	94.0	98.2
**Accuracy**,** %**	90.7	88.5	95.7	90.6	96.0	89.4	88.4	78.7	97.0	90.6
**AUC**	0.926	0.876	0.962	0.912	0.900	0.850	0.850	0.797	0.971	0.917

^a^: PPV, positive predictive value, the number of true-positive patients divided by the number of all patients who test positive.

^b^: NPV, negative predictive value, the number of true-negative patients divided by the number of all patients who test negative.

**Fig. 1:**
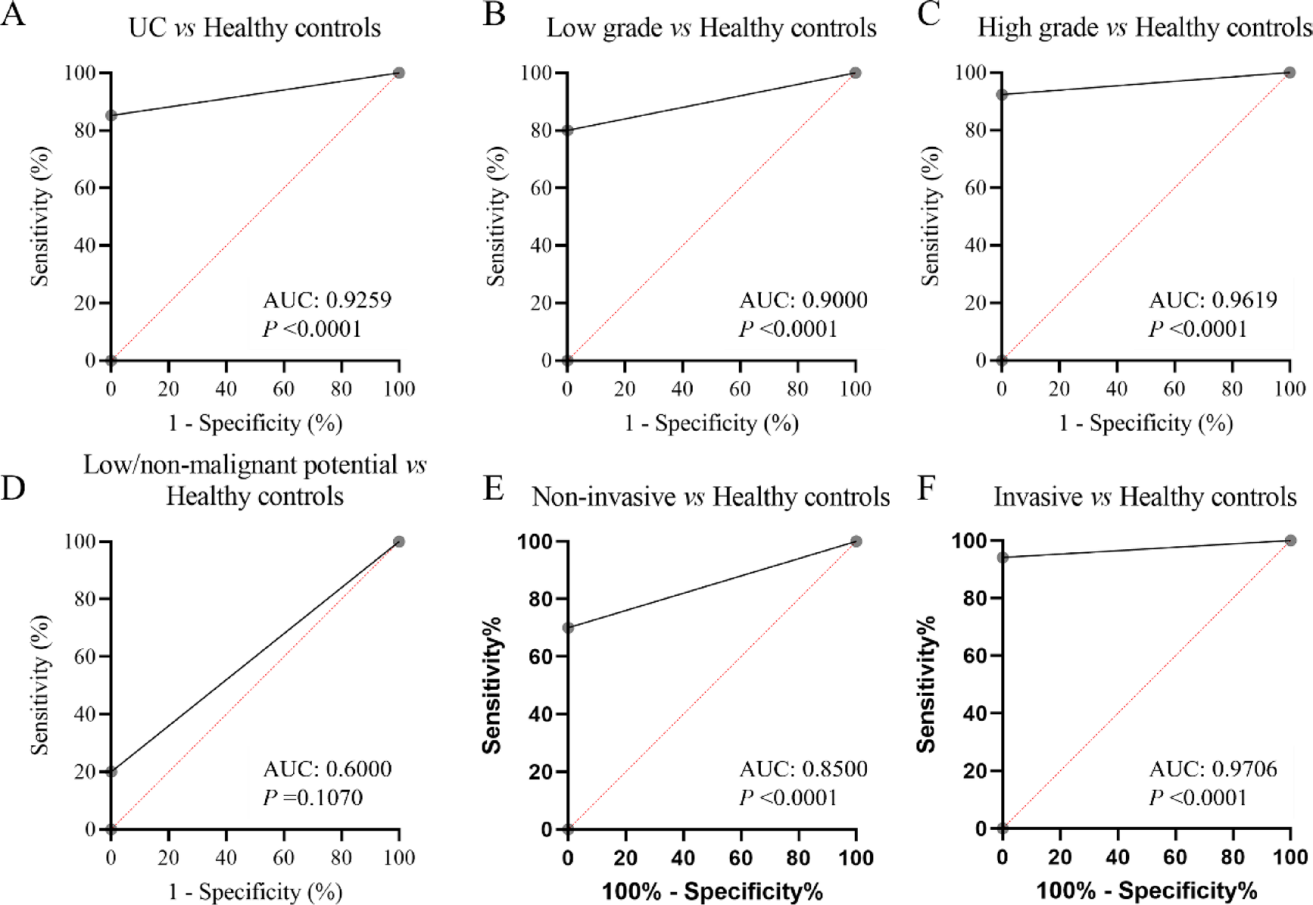
The diagnostic potential of the two-gene DNA methylation assay for UC. (**A**) The ROC curve comparing UC patients and healthy controls. (**B**) The ROC curve comparing low grade UC patients and healthy controls. (**C**) The ROC curve comparing high grade UC patients and healthy controls. (**D**) The ROC curve comparing low grade low/non-malignant potential UC patients and healthy controls. (**E**) The ROC curve comparing non-invasive UC patients and healthy controls. (**F**) The ROC curve comparing invasive UC patients and healthy controls.

Firstly, the assay’s performance in distinguishing UC patients from healthy individuals was evaluated. The results showed no positive samples in healthy volunteers, while the positive rate of methylation reached 85.2% in UC patients (Supporting Table 2). The AUC of this receiver operating characteristic curve (ROC) reached 0.9259, with a sensitivity (SE) and specificity (SPE) of 85.2% and 100% (Fig. 1A; Table 2). Further analyses comparing different stratification of UC patients with healthy controls were performed. In low grade UC patients versus healthy controls group, the AUC was 0.9000, with SE and SPE of 80.0% and 100%. While in high grade UC patients versus healthy controls group, the AUC was 0.9619, with SE and SPE of 92.4% and 100% (Table 2; Fig. 1B, C). Of note, the positive rate of methylation was only 20% in low/non-malignant potential UC patients, implying a strong association between the two-gene methylation and tumor malignancy (Fig. 1D). Moreover, when UC patients were stratified into non-invasive and invasive disease, the diagnostic ability was also significant (non-invasive vs. healthy: SE 70.0%, SPE 100%, AUC 0.8500; invasive vs. healthy: SE 94.1%, SPE 100%, AUC 0.9706, Table 2; Fig. 1E, F).

The assay’s performance in distinguishing UC patients from non-UC urologic diseases was further evaluated. The positive rates of two-gene DNA methylation were between 2.6 and 20.0% in non-UC urologic diseases (Supporting Table 2). The SE, SPE and AUC of this assay for distinguishing UC from non-UC urologic diseases were 85.2%, 90.0% and 0.876, indicating the superior diagnostic ability to differentiate UC from non-UC urologic diseases (Table 2).

**Fig. 2:**
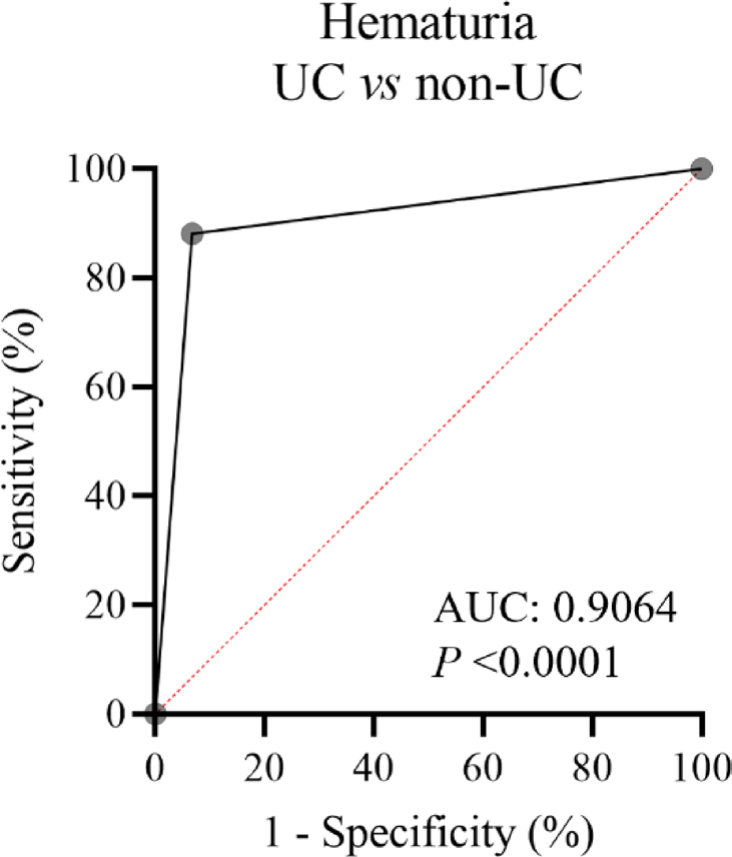
The ROC curve comparing at risk patients of UC and non-UC.

In clinical practice, hematuria is the classic symptom and the first sign of UC but also seen in other urinary disorders. The performance of using hematuria alone to diagnose UC is low (sensitivity 56%, specificity 89.9%, AUC 0.74). Because UC is a relatively severe condition compared to others, it is of great importance to screen out UC diagnoses from patients with hematuria, achieving early intervention. Therefore, the assay’s performance in identification of UC patients from patients with hematuria was further evaluated. Among patients mentioned above, hematuria was presented in 126 (93.3%) of UC patients and 132 (43.9%) of non-UC patients. In the presence of hematuria, the diagnostic performance of the assay is even more prominent, with a SE of 88.1%, SPE of 93.2% and AUC of 0.9046 (Fig. 2). Taken together, our data indicated that the DNA methylation assay can be served as a diagnostic tool for UC.

### Correlation between the DNA methylation assay and UC severity

The correlation between the DNA methylation and UC characteristics was further investigated. Results indicated that higher positive rate of the DNA methylation assay correlated with higher tumor grade (positive rate: low malignant potential 20%, low grade 80% and high grade 92.4%, *p* < 0.0001, Table 3) and invasiveness (positive rate: non-invasive 76.2% and invasive 93.1%, *p* = 0.0074, Table 3).

**Table 3:** The association between the methylation and the clinicopathological features of UC patients.

	Urothelial carcinoma(*n* = 135)			
	Overall	Positive	Negative	*P* value
**Age/year (Median(range))**	68 (31–91)	65 (33–91)	66 (31–83)	
**Gender**				
Male (%)	110 (81.5%)	90 (78.3%)	20 (100%)	0.0245^#^
Female (%)	25 (18.5%)	25 (21.7%)	0 (0%)	
**WHO 2004/2016**				
Low grade	20 (14.8%)	16 (13.9%)	4 (20.0%)	< 0.0001
High grade	105 (77.8%)	97 (84.3%)	8 (40.0%)	
Low/non-malignant potential	10 (7.4%)	2 (1.7%)	8 (40.0%)	
**TNM Stage**				
0	13 (9.6%)	12 (10.4%)	2 (10.0%)	0.2682
I	50 (37.0%)	66 (57.4%)	16 (80.0%)	
II	52 (38.5%)	17 (14.8%)	2 (10.0%)	
III	10 (7.4%)	10 (8.7%)	0 (0%)	
IV	10 (7.4%)	10 (8.7%)	0 (0%)	
**T Stage**				
Ta	13 (9.6%)	11 (9.6%)	2 (10%)	0.2967^$^
Tis	0 (0%)	0 (0%)	0 (0%)	
T1	50 (37.0%)	67 (58.3%)	16 (80.0%)	
T2	54 (40.0%)	19 (16.5%)	2 (10.0%)	
T3	11 (8.1%)	11 (9.6%)	0 (0%)	
T4	7 (5.2%)	7 (6.1%)	0 (0%)	
**Invasiveness**				
Non-invasive	50 (37.0%)	35 (30.4%)	15 (75.0%)	0.0074^#^
Invasive	85 (63.0%)	80 (69.6%)	5 (25.0%)	

#: Fisher’s exact test.

$: Tis excluded.

### Assay performance in detecting UC recurrence

**Fig. 3:**
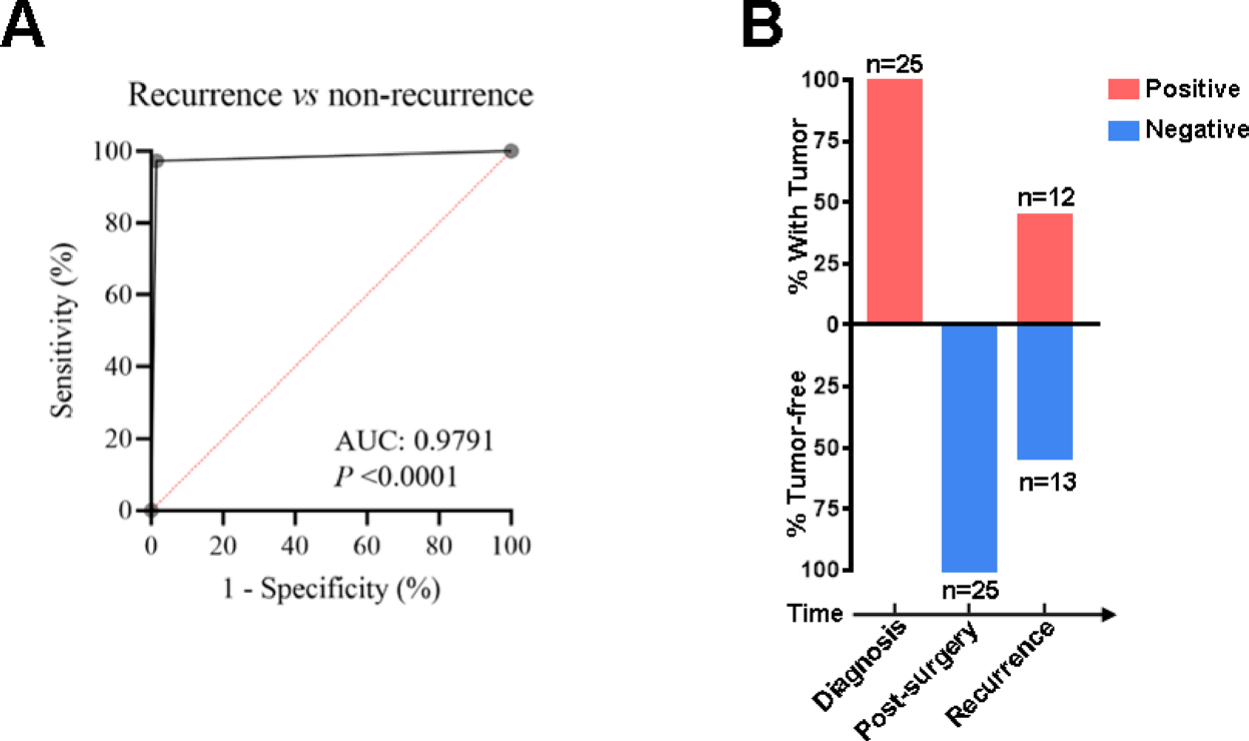
Assay performance in detecting UC recurrence. **(A)**The ROC curve comparing post-surgery UC patients with/without recurrence. **(B)**The percentage of positive/negative DNA methylation assay of UC patients at diagnosis, post-surgery and recurrence.

To verified the assay’s performance in recurrence monitoring, a total of 179 follow-up patients diagnosed with UC, who had undergone transurethral resection, were additionally recruited. Their urine samples were collected before routine follow-up surveillance cystoscopy and analyzed using the DNA methylation assay. Among 67 patients with UC recurrence, the assay detection had a positive rate of 98.5%, while among 112 patients without recurrence, the positive rate was only 2.7%. The AUC of ROC was 0.9791, demonstrating superior recurrence detection ability (Fig. 3A; Table 4). Additionally, out of the total participants, 25 UC patients tested methylation positive before surgery and became negative after radical cystectomy. Moreover, 13 of them eventually replased and tested positive with the assay again, while the other 12 patients had no recurrence and remained negative in the DNA methylation assay (Fig. 3B; Table 5). These results suggested that the DNA methylation assay had great potential for use in monitoring recurrence of post-surgery UC patients.

**Table 4:** The positive rate of UC patients with/without recurrence after surgery.

	Recurrence (*n* = 67)	Non-recurrence (*n* = 112)
**DNA methylation assay**		
Positive	66	3
Negative	1	109
Positive rate	98.5%	2.7%

**Table 5:** The positive rate of UC patients with/without recurrence during follow-up after radical cystectomy.

	Recurrence (*n* = 13)	Non-recurrence (*n* = 12)
**DNA methylation assay**		
Positive	13	0
Negative	0	12
Positive rate	100.0%	0.0%

## Discussion

UC is a disease that requires systemic and multi-approach management. Current gold standard for UC screening is still cystoscopy, which is invasive and not cost-effective. Recently, urine DNA methylation detection has been extensively studied in UC diagnosis due to its inexpensive, painless, and reproducible nature^15,16^. The detection of DNA methylation targeting the genes HIST1H4F and SOX1-OT of our assay in both tumor tissues and urine samples from UC patients suggests the exfoliated-cell origin and tumor specificity of the urinary DNA methylation.

Different studies of DNA methylation have been conducted, and many targets are proven to be applicable in bladder UC diagnosis. For example, one research detects the DNA methylation status of TWIST1, ONECUT2 (2 loci) and OTX1 in urine to diagnose bladder cancer among hematuria patients. The AUC of their test reached 0.93 when combining with other variables including mutation status of FGFR3, TERT and HRAS and age^19^. However, mutation status is difficult to detect, making this test less cost-effective. In another research on urine DNA methylation and bladder cancer detection, the authors established a diagnosis model consisting of 8 markers, which achieved SE and SPE of 0.83 and 0.6^21^. But this model had too many markers, which may limit its clinical application. In another research with bladder UC diagnosis purpose, the authors used a kit called EpiCheck, which detected 15 methylation markers in urine, to screen for recurrence in a cohort of 353 postsurgical bladder UC patients. The AUC of this kit is 0.82. But if low-grade patients were excluded, the AUC could reach 0.94^14^.

In our study, results demonstrate the potential of the DNA methylation assay based on HIST1H4F and SOX1-OT combination in detecting and distinguishing UC from healthy individuals and patients with non-UC urologic diseases. Previous researches had studies these two markers separately in bladder UC. HIST1H4F was shown to be hypermethylated on its exon regions, and the AUC of the diagnosis ROC curve (bladder UC vs. healthy controls) was 0.889. Their results also demonstrated that the methylation did not have an effect on HIST1H4F RNA expression. However, their assay was based on tissues, but not urine, limiting its application in non-invasive detection^17^. SOX1-OT was studied in another research, which was one of the two markers in their diagnostic model for bladder UC. Their model used urine as samples, had a relatively high diagnostic power (AUCs were 0.919 and 0.903 in train and test cohort), and validated samples from 5 different medical centers^15^. We re-tested SOX1-OT in our samples and decided combine it with HIST1H4F to establish our diagnostic model. Our assay showed high sensitivity and specificity in identifying UC patients, even in different subgroups based on tumor grade, invasiveness, and malignant potential. This indicates the robustness of the assay in detecting UC across various clinical scenarios. Furthermore, the correlation analysis between the DNA methylation assay results and UC characteristics such as tumor grade and clinical stage suggests a relationship between the assay’s positive rate and the severity of the disease. The higher positive rates observed in high-grade tumors and advanced stages of UC support the potential utility of the assay in assessing disease severity and progression. But as a binary diagnostic test, this trend only applies to theoretical analyses. In clinical practice, the focus of this test still lies in diagnostic purpose, and other multimodal methods should be used in order to determine the severity of UC patients. Moreover, the performance of the DNA methylation assay in detecting UC recurrence highlights its potential for post-surgery surveillance in UC patients. The high positive rate among patients with recurrent UC and the ability to detect recurrence before clinical manifestation suggest that the assay could be a valuable tool in monitoring disease recurrence and guiding treatment decisions in a timely manner. Overall, our findings suggest that the DNA methylation assay based on the two-gene combination has promising diagnostic and prognostic value in the management of UC. Further validation studies with larger patient cohorts are warranted to confirm the utility of this assay in clinical practice and its potential integration into routine follow-up protocols for UC patients.

Although hematuria can be indicative of various urinary disorders, including but not limited to UC, the presence of hematuria in a large proportion of UC patients underscores its importance as a clinical indicator for further evaluation. Our results showed the superior diagnostic performance of the DNA methylation assay in the presence of hematuria, highlighting the potential synergistic effect of combining these two biomarkers for UC detection. By integrating hematuria assessment with DNA methylation analysis, clinicians may enhance the diagnostic accuracy and specificity in identifying UC cases. The combination of these two diagnostic modalities may offer a more comprehensive approach to differentiate UC from other benign urological conditions and non-UC urologic diseases. Future studies focusing on the combined use of hematuria and DNA methylation in larger patient cohorts can further validate the utility of this approach in enhancing the early detection and management of UC. Also, urine cytology, which is a routine clinical practice during the diagnosis of UC, can be combine with our methylation test to further enhance the overall accuracy of diagnosis.

Our study proposed a DNA methylation assay that could be further developed into a diagnostic method, however, there are still several limitations existing in our study. First, the patient number of our cohort is not big enough, and also the cohort is from single center. This may result in lower statistical significance. Second, our study did not screen methylation markers with sequencing method, instead, two markers were summarized and picked with review of previous literatures and validation with patient tissues from our hospital. Although the assay demonstrates high diagnostic power, it still lacks novelty in terms of discovering new markers for bladder UC. Third, our study only performs clinical analysis, but the actual mechanism of HIST1H4F and SOC1-OT on bladder malignancy is still largely unknown. Further molecular experiments are needed to fully elucidate the role of these two markers in bladder tumorigenesis.

Although our study suggests that HIST1H4F and SOC1-OT are hypermethylated in UC tumors, whether such methylation has an impact on tumor progression remains unknown. So far, few studies have reported the function and role of these two genes in tumors. Further studies should focus on elucidating the functional significance of HIST1H4F and SOX1-OT hypermethylation in UC tumorigenesis, growth, invasion, and metastasis to understand their potential role in tumor progression. Investigating the molecular mechanisms underlying the methylation of these genes and their impact on key pathways involved in UC pathogenesis could provide valuable insights into the biological significance of these epigenetic alterations. Furthermore, understanding the biological functions of these genes in the context of UC could pave the way for the development of targeted therapies or personalized treatment strategies based on the specific molecular profiles of individual tumors.

## Conclusion

This research proposes a novel, non-invasive DNA methylation assay using urine as sample for detection of bladder UC. Validations with cohort from our institute indicated that this assay had high power in diagnosis and recurrence surveillance of bladder UC. This assay has the potential to be developed into an applicable method for clinical practice, however, further study is needed to fully address issues including external validation and molecular mechanism.

## Supplementary Information

Below is the link to the electronic supplementary material.

Supplementary Material 1

Supplementary Material 2

## Data Availability

For all data requests, please contact the corresponding author.
