# Supplementary material for: A urinary DNA methylation assay using two genes enables noninvasive detection and prognostic prediction in urothelial carcinoma
Source: Sci Rep. 2025 Aug 25;15:31281. doi: 10.1038/s41598-025-14646-0 (PMC12378184; doi:10.1038/s41598-025-14646-0)
Supplement: Supplementary file 1 — Supplementary Material 1 [file 41598_2025_14646_MOESM1_ESM.docx]

**Supporting Table 1**. Primers used in this research

| gene ID | probe ID /genomic location | Forward primer | reverse primer | probe |
| --- | --- | --- | --- | --- |
| HIST1H4F | cg10723962 | CTCCGTATAAATTACGACGTCCC | GGCGGCGTGAAAC | CGGGTTTTATTTATGAGGAGATTC |
|  | cg21425842, cg08260959 | TTATACGGAGTACGTTAAGCG | AAAACTCAACCACCAAAACCG | CGCGTTTAAGCGTTAGGGACG |
| SOX1-OT | cg11437784 | GTAGGTTAGGTCGGAACGCG | CCATTCGCTCGCTCTC | GGGGGCGCGGGAGGGGGATTC |
|  | cg15736169, cg03898631 | CGGTTATTTAGGTAGAACGGGGT | CGTAACGACCGAAAATCCGAAC | ACGCAACTACTCGACGCTTTAAACGCCCG |
| NRN1 | cg11564981 | TAAATTAAATTTTCGGGGTTTGGGC | GCCAACCGCGCCGAACG | CGTTCGCGTGTTTTGTGTGCGGGC |
| POU4F2 | cg02610222 | ATAGAGTTCGGAGGCGGCGGC | CGCCGAAACGCTAATCCG | AAAAATACCGAACCAACCG |
|  | cg24199834 | CGAGGTTTGTAGTTAGC | TCGACTACGCGCTCCTTCG | CGAAGGAGCGCGTAGTC |
| Vim | cg11973177 | CGTGGTGTTATCGGATTTTTTTGGT | AACTAAAACTTTTAAAAACTTTCCGAA | TAAAATACTAAAAAAAACGAAATCGC |
|  | NC_000010.11: 17229406..17229407 | TTCGGGAGTTAGTTCGCGTT | ACCGCCGAACATCCTACGA | TCGTTGGTCGACGTTATTAATATC |
| Onecut2 | cg02250594 | CGGCGTCGCGTTCGG | CGAATCGCAAAACATACTCATAACGTA | ACGGCGGCGATTATCGGTTCGAGT |
|  | NC_000018.10: 57435830..57435831 | AACCAACAACTCCTACTCATAACCC | GTTTTCGTCGTTTTCGGGTTTTGATG | ACTACCGCCGCCGACCGACC |
| Twist1 | cg26818735, cg26312150 | CGTTTTCGTACGTTGGTTATGATTC | CGACGACGCGAACGACGAC | CGTAAGATTGCGGATTTTCGTCGTC |
