# Supplementary material for: A urinary DNA methylation assay using two genes enables noninvasive detection and prognostic prediction in urothelial carcinoma
Source: Sci Rep. 2025 Aug 25;15:31281. doi: 10.1038/s41598-025-14646-0 (PMC12378184; doi:10.1038/s41598-025-14646-0)
Supplement: Supplementary file 2 — Supplementary Material 2 [file 41598_2025_14646_MOESM2_ESM.docx]

**Supporting Table 2**. Features and positive rate of three groups of patients tested with the methylation assay

|  | **Urothelial carcinoma（n=135）** | **Non-Urothelial carcinoma** | | | | **Healthy controls(n=79)** | ***P* value** |
| --- | --- | --- | --- | --- | --- | --- | --- |
|  |  | **Clear cell renal cell carcinoma(n=39)** | **Prostate cancer(n=45)** | **Other tumors of the urinary system(n=161)** | **Benign urological conditions(n=56)** |  |  |
| **Age/year (Median(range))** | 68 (31-91) | 53 (33-65) | 68 (47-87) | 58 (14-74) | 48 (25-86) | 63 (22-82) | NS |
| **Gender (amount (proportion))** | |  |  |  |  |  |  |
| Male | 110(81.5%) | 24 (61.5%) | 45 (100%) | 85 (52.8%) | 30 (53.6%) | 61 (77.2%) | NS |
| Female | 25(18.5%) | 15 (38.5%) | 0 (0%) | 76 (47.2%) | 26 (46.4%) | 18 (22.8%) |  |
| **Hematuria positive** | 126(93.3%) | 132 (43.9%) | | | | - | *** |
| **DNAmethylation assay** |  |  |  |  |  |  |  |
| Positive | 115 | 1 | 9 | 20 | 2 | 0 | *** |
| Negative | 20 | 38 | 36 | 141 | 54 | 79 |  |
| Positive rate | 85.2% | 2.6% | 20.0% | 12.4% | 3.6% | 0.0% |  |
